# Postpercutaneous Nephrolithotomy Nephrostogram: Is It Mandatory? A Single Center Experience

**DOI:** 10.1155/2014/423730

**Published:** 2014-02-03

**Authors:** Abdul Rouf Khawaja, Tanveer Iqbal Dar, Ajay Kumar Sharma, Farzana Bashir, Vipin Kumar Tyagi, Mohammad Sajid Bazaz

**Affiliations:** Department of Urology, Sir Ganga Ram Hospital, New Delhi 110060, India

## Abstract

*Aims and Objective*. “Postpercutaneous nephrolithotomy nephrostogram” (PPNN) is routinely performed in most of the centers. No published series could be found in the literature without post percutaneous nephrolithotomy nephrostogram. Hence, the aim of our study is to highlight that post percutaneous nephrolithotomy nephrostogram is not mandatory and it only adds to cost and morbidity without adding any information in the management of such patients. *Methods*. It was a prospective study from 2005 to 2012, conducted in our institute. It included 119 patients of renal stones who underwent percutaneous nephrolithotomy performed under the guidance of a single surgeon. Postoperative nephrostogram was not done in any of the patients. *Results*. Complete stone clearance was achieved in 97.5% of patients and 2.5% of patients needed two to three sessions of ESWL later on. None of the patients needed second look percutaneous nephrolithotomy or nephrostogram. *Conclusion*. Postpercutaneous nephrolithotomy nephrostogram increases chances of infection, inconvenience, contrast related complications, and cost, with no added advantage over plain X-ray KUB, and it should not be done as a routine investigation prior to the removal of PCN tube in patients with complete stone clearance.

## 1. Introduction 

The revolution regarding percutaneous access started way back in 1955 when Goodwin and his associates did the first percutaneous nephrostomy, a tract meant for drainage of pus and urine. In late 1970 this access was further utilized for removal of stones; initially it was for high risk patients and done at specialized centers, but over the years it has been practiced in many centers and has replaced open operation in the majority of patients with renal stones. Percutaneous nephrolithotomy (PCNL) is the preferred treatment for large, >2 cms, renal or stag horn renal stones [1]. The planning and successful execution of the initial puncture into the kidney are crucial to the outcome of PCNL. The urologist's selection of the optimum tract based on the intrarenal anatomy and the ability to make secondary tracts as required permit more effective stone removal [2]. PCNL procedure has advantage of short hospital stay, small stab wound scar, and negligible pain in the postoperative period [2]. There are only few centers where post-PCNL nephrostogram is not a routine.

## 2. Material and Methods

This was a prospective study conducted in Sir Ganga Ram Hospital, New Delhi. A total of 119 renal stone patients were included who underwent percutaneous nephrolithotomy (PCNL) performed by a single surgeon from 2006 to 2012, in which nephrostogram was not performed as a routine prior to the removal of nephrostomy tube. Patients with concomitant ureteral stones/strictures and solitary kidney with stones were excluded.

All patients underwent detailed history, clinical examination, urinalysis/urine culture, haemogram and kidney function tests, ultrasound kidney ureter bladder (KUB), intravenous urography, and computed tomogram (CT) urography in selected cases, before the procedure. For all patients the procedure was explained and was written informed consent obtained. Renal stones, unilateral or bilateral exceeding two centimeters, were included. Patients whose urine culture was positive were treated by antibiotics for at least two weeks prior to the procedure. All the patients underwent PCNL performed by the same surgeon.

All patients underwent cystoscopic examination with retrograde pyelography (RGP) before ureteric catheterization and distal patency of ureter was assessed. The open ended ureteric catheter (5 or 6 French) was then passed up to renal pelvis and fixed with Foley catheter. Patient was then turned prone for PCNL. Laparoscopic assisted percutaneous transperitoneal PCNL was done in ectopic kidneys.

The desired posterior calyx (middle, superior, or inferior calyx, depending on position of stone) was punctured by 18 gauge needles and was tract dilated by Amplatz dilators under fluoroscopic control, up to 26–30 French (Fr), depending on the stone burden. An Amplatz sheath was passed over the last dilator. Pneumatic lithotripsy was used to break stone into manageable fragments. Small fragments in inaccessible calyces were pushed into the desired calyx by injecting saline jet under fluoroscopic guidance through a different puncture into that calyx (without dilating it). After complete clearance of fragments 14 Fr Melicort nephrostomy tube (PCN) was indwelled and intraoperative nephrostogram was done for any residual fragments and patency of the ureter. At the completion of procedure the open ended ureteric catheter was left in situ. Those with extravasation of contrast were stented by double J stent which was removed after 4 to 6 weeks. Foley catheter along with the ureteric catheter was removed on first postoperative day in all other patients after getting a fresh X-ray KUB region done for any missed residual calculi. The PCN tube was then clamped for two to three hours before taking it out. In those who developed acute colicky pain or significant urine leak, the tube was reopened and removed in all others. Those who developed significant urine leak or severe colicky pain were discharged on PCN tube which was removed after 48 hours in outpatient department (OPD). None of the patients underwent nephrostogram prior to the removal of PCN tube.

## 3. Observations

A total of 119 patients were recruited in the study demographic and clinical profiles are shown in Table 1. Age ranged from 17 to 64 years (mean 37). Males predominated with male-female ratio 2.6 : 1 (86 : 33). Average stone size was 2.8 cm; complete stag horn calculus was seen in 27 patients and bilateral stones were seen in 13. Two patients were operated on for calculi in pelvic kidney by laparoscopically assisted PCNL. The procedure was uneventful in all with blood transfusion needed in 3 (average 300 mL packed red blood cells) and angioembolization of the bleeder in one patient. Complete stone clearance was achieved in 116 patients (97.5%), with significant residual fragments in 3 which were cleared by ESWL later on. The procedure of PCNL was abandoned in them because of torrential bleeding. Three patients complained of mild colicky pain in respective flank. Residual insignificant fragments of 3–5 mm size were seen in them (two in upper and one in lower ureter) in unenhanced CT scan within the first week of surgery. These fragments passed spontaneously and were missed by the postoperative X-ray KUB. Extravasation of contrast was seen in 13 (15.4%) patients during on table postoperative nephrostogram under fluoroscopy. These 13 patients were stented by DJ stent which was removed after 4 to 6 weeks.

All the patients were followed after one week and sutures were removed. Transient mild leak from nephrostomy site was seen in 10 (10.9%) patients after nephrostomy removal which settled within 24–48 hours.

## 4. Discussion

With advances in modern medicine, extracorporeal shock wave lithotripsy, retrograde intrarenal surgery (RIRS), and PCNL have become the treatments of choice for all cases of renal calculus disease [1]. Percutaneous nephrolithotomy (PCNL) is the preferred modality of management for stones more than 2 cm in size. As compared to open surgical procedures, ESWL and PCNL are less invasive and have lower complication rates and a significantly shorter convalescent phase [2–4]. Economics are an important consideration in developing countries with a high burden of stone disease. PCNL is much less invasive than open surgery and stone free rates of 98 to 99% can be achieved [3]. In addition there is a significant reduction in the cost of treatment [3]. As the size of the stone increases and as the complexity of the situation increases, the stone free rate drops to 75–85%. Better results are achievable with greater effort, and it becomes a matter of judgment as to whether a given residual stone is worth the effort required to remove it [3].

Nephrostomy tube provides adequate renal drainage, allowing renal healing and avoiding urinary extravasation. It may also tamponade bleeding and allow the nephrostomy tract to mature and make second look nephroscopy easier [5]. We had nephrostomy site leak in 10.9% of patients which settled spontaneously. In our study complete clearance of stones was achieved in 116 (97.5%) patients with significant residual fragments in kidney in 3 and clinically insignificant fragments in ureter in 3 patients detected by CT scan. 10 patients (10.9%) had mild leak which settled within 48 hours. The success of PCNL depends on meticulous technique and experience. Inevitably, experience and time are required, with improving results over time. Halachmi et al. [6] in a study concluded that unenhanced multidetector CT is more accurate than antegrade pyelography via a PCN tube for the assessment of urinary tract stones, with the advantage of reducing the risks of contrast injection and picking up small fragments which are missed by routine nephrostogram. We did not perform nephrostogram in any of our patients as we did not believe it would help us, and we confirmed that supposition. In a study on “diagnostic utility of post-PCNL nephrostogram” Andonian et al. concluded that while distal obstruction seems to predict prolonged urinary drainage (>24 hours) it may not necessitate placement of ureteral stent or prolonged nephrostomy drainage because blood clot and UVJ edema resolve spontaneously with expectant management [7]. Hence, the management will not alter by doing or omitting the nephrostogram, adding to the cost, inconvenience, and infective episodes.

PCNL has been used successfully in patients with renal failure secondary to stone disease with little deleterious effect on the renal function. Indeed, a significant improvement was documented for impaired renal function with the removal of stones and clearance of infection [8–11]. PCNL is a safe and effective method of stone removal in patients with calculi in horseshoe kidneys [12]. In our study 2 cases of pelvic kidney were also included where the stone was removed with laparoscopic assisted transperitoneal posteriorly placed upper or middle pole puncture and successful stone removal was achieved. Sometimes stones are extruded through the collecting system or are noted in the perinephric tissues outside the kidney. It is not necessary to remove these stones, as experience has shown them to be clinically unimportant. Their main importance has been to generate confusion on subsequent plain abdominal radiographs.

It is important to note that antegrade nephrostogram will at times be an important postoperative study, specifically if one is evaluating for urinary extravasation, adequate positioning of the nephrostomy tube, residual ureteral obstruction unrelated to calculus, or adequacy of access for a second-look procedure [6]. However, in our study we did not need second look surgery, neither did we include patients with preoperative ureteral obstruction. Also the patients with urinary extravasation were stented for 6 weeks, again not needing nephrostogram.

## 5. Conclusion

Post-PCNL nephrostogram need not be done as a routine investigation prior to removal of PCN tube in patients with complete stone clearance. It leads to inconvenience contrast related complications and adds to the cost, with no added advantage over plain X-ray KUB.

## Figures and Tables

**Table 1:** Clinical profile of the patients (total 119).

Total patients	119
Age	17–64 years
Male/female ratio	2.6 : 1
Unilateral/bilateral PCNL	106/13
Residual stone	3
ESWL	3
Angioembolization	1
Extravasation/DJS	13/13
Leak	10 (8.4)
Nephrostogram/NCCT	0/3

ESWL: extracorporeal shockwave lithotripsy, DJS: double j stent, and NCCT: noncontrast computed tomogram.
